# Mechanical Properties of Additively Manufactured Thermoplastic Polyurethane (TPU) Material Affected by Various Processing Parameters

**DOI:** 10.3390/polym12123010

**Published:** 2020-12-16

**Authors:** Tao Xu, Wei Shen, Xiaoshan Lin, Yi Min Xie

**Affiliations:** 1 Centre for Innovative Structures and Materials, School of Engineering, RMIT University, Melbourne 3001, Australia; s3789870@student.rmit.edu.au (T.X.); susanna.lin@rmit.edu.au (X.L.); 2 Ameba Engineering Structure Optimization Research Institute, Nanjing 211800, China; william.shen@xieym.com

**Keywords:** thermoplastic polyurethane, TPU, selective laser sintering, mechanical properties, build orientation

## Abstract

Thermoplastic polyurethane (TPU) is a polymer material that has high ductility, good biocompatibility and excellent abrasion resistance. These properties open a pathway to manufacturing functional TPU parts for applications in various fields such as aerospace engineering, medical devices and sports equipment. This study aims to investigate the mechanical properties of additively manufactured TPU material affected by three different processing parameters, including build orientation, mix ratio of the new and reused powders and post-processing. A series of material tests are conducted on TPU dumb-bell specimens. It is found that the mix ratio of the new powder is the most critical factor in improving the mechanical properties of the printed TPU parts. Compared to reused powder, new powder has better particle quality and thermal properties. Besides, build orientation is also a very important factor. TPU parts printed in flat and on-edge orientations show better tensile strength and deformability than those printed in upright orientation. In addition, post-processing is found to significantly enhance the deformability of TPU parts.

## 1. Introduction

**Figure 1:**
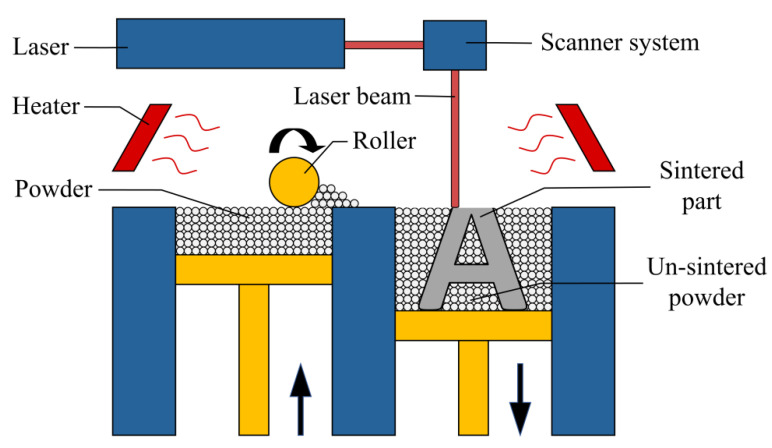
Schematic of selective laser sintering.

Additive manufacturing (AM) technologies have many advantages over traditional manufacturing methods [1]. They could significantly reduce the design and production cycle by using rapid prototyping [2]. More importantly, they enable the production of parts with highly complex geometries, such as those generated from a topology optimization process [3,4,5,6,7,8]. Selective laser sintering (SLS) is one of the most widely used AM technologies, which employs a laser to sinter powder layer by layer to form functional end-use components [9,10,11,12]. The schematic of SLS is shown in Figure 1. SLS has been increasingly employed to manufacture functional parts in various areas, including aerospace [13], medical engineering [14,15] and sports [16].

Thermoplastic polyurethane (TPU) is an additively manufacturable polymer material that has high ductility, good hydrolysis resistance, excellent biocompatibility and great abrasion resistance [17,18,19,20,21,22,23,24]. It can be used in structures that require high ductility, such as energy-absorbing structures [25,26] and wearable devices [27,28]. In the past, the effect of TPU powder on the mechanical properties of printed parts has been studied by many researchers. Dadbakhsh et al. [29] investigated the influences of the size and shape of TPU powder on SLS processability and mechanical properties of printed TPU parts. The effects of powder bed temperature, laser energy density, powder particle size and powder composition on the properties of laser-sintered TPU were studied by Gan et al. [30]. The powder flow, rheology of melt, shrinkage and hardening behavior involved in TPU sintering through the analysis of four different TPU grades were examined by Verbelen et al. [31]. A systematic evaluation of the physical and thermal properties of TPU powder for SLS was presented by Yuan et al. [32]. In all these studies, the mechanical properties of additively manufactured TPU parts were found to be highly dependent on the powder quality, especially the size and thermal behavior of particles.

Build orientation also has a significant influence on the mechanical properties of additively manufactured parts. Barba et al. [33] studied the effect of build orientation on the mechanical properties of additively manufactured metal parts. Chacón et al. [34] characterized the effect of build orientation on PLA samples. However, the effect of build orientation on the additively manufactured TPU parts has not been studied previously.

Due to the powder particle size and layer-by-layer process, SLS parts inherently exhibit a rough and grainy surface finish [35]. The surface finish could be improved by chemical treatment. The chemical solvent could react with part of the surface to form a coating, thereby improving the corrosion resistance and extending the service life of the product [36]. Previously, researchers have been focused on the adhesion of polymer materials after chemical treatment [37,38], and very few studies have been reported on the effect of post-processing on the mechanical properties of printed parts. However, it shall be noted that the composition of the printed part may be changed by chemical treatment, leading to the changes of mechanical properties.

In addition, during the SLS process, the un-sintered powder is exposed to field temperature and serves as support for the printed parts (Figure 1). In order to save costs, after removing the printed part, the un-sintered powder is generally mixed with new powder in a particular proportion for the next processing round [39]. So far, to the best of the authors’ knowledge, no study has reported on the influence of the mix ratio of the new powder and reused powder on the mechanical properties of sintered parts.

Based on the above discussion, in this study, a series of experimental tests are conducted on TPU dumb-bell specimens manufactured with an industrial SLS printer. The effects of three processing parameters on the mechanical properties of 3D printed TPU parts are investigated, including build orientation, post-processing and mix ratio. Besides, the microstructure and the thermal behavior of TPU powder are characterized based on the test results obtained from scanning electron microscope (SEM) and differential scanning calorimetry (DSC). The findings of this research could provide clear guidelines for the selection of processing parameters of additively manufactured TPU parts.

## 2. Materials and Methods

### 2.1. Test Specimens

**Figure 2:**
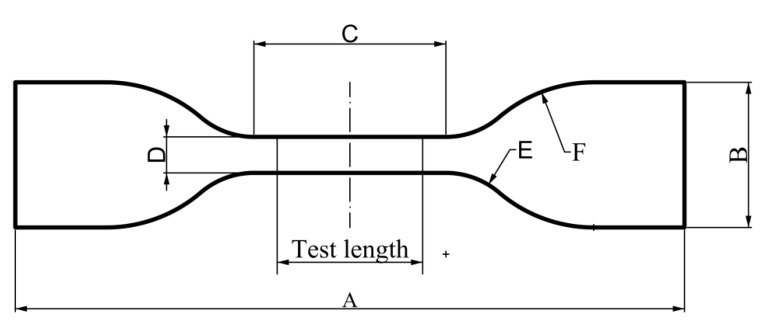
Shape of the dumb-bell test specimens [40].

**Figure 3:**
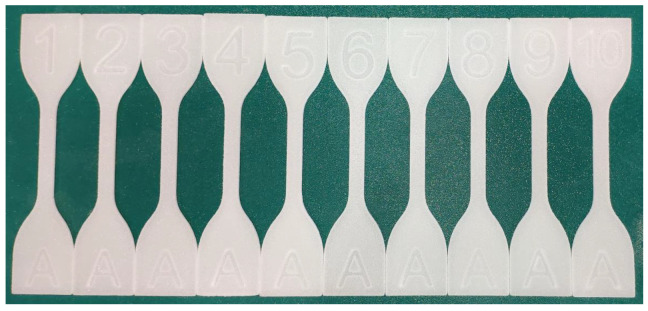
3D printed dumb-bell test specimens.

In this study, all specimens were printed using an industrial SLS machine (Farsoon Technologies-HS403, Changsha, China) with the TPU powder type WANFAB-PU95AN. According to ISO37 standard (Rubber, vulcanized or thermoplastic—Determination of tensile stress–strain properties) [40], the outline of the dumb-bell pieces for tensile tests is shown in Figure 2. The dimensions of the dumb-bell are summarized in Table 1. Figure 3 shows the 3D printed dumb-bell specimens.

### 2.2. Processing Parameters

The processing parameters investigated in this study included build orientation, post-processing and mix ratio, which are listed in Table 2. In this study, the parameter settings on the SLS machine were consistent for all test specimens. The laser power was 55 W. The scanning speed and the scanning space were set at 15.2 mm/s and 0.1 mm, respectively.

#### 2.2.1. Build Orientation

**Figure 4:**
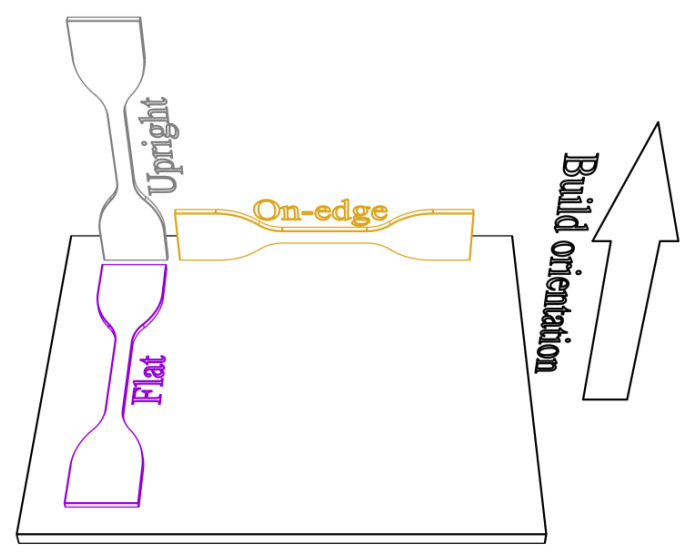
Three build orientations.

Build orientation refers to which orientation a printed part is placed on the print platform. Thus, the printed part may show an anisotropic property, and it is essential to analyze the effect of orientation on the mechanical properties of 3D printed TPU parts. In this study, three build orientations were assessed: flat, on-edge and upright (Figure 4). The flat specimens were printed along the thickness direction, whereas the on-edge and upright specimens were printed along the width and length directions, respectively.

#### 2.2.2. Post-Processing

**Figure 5:**
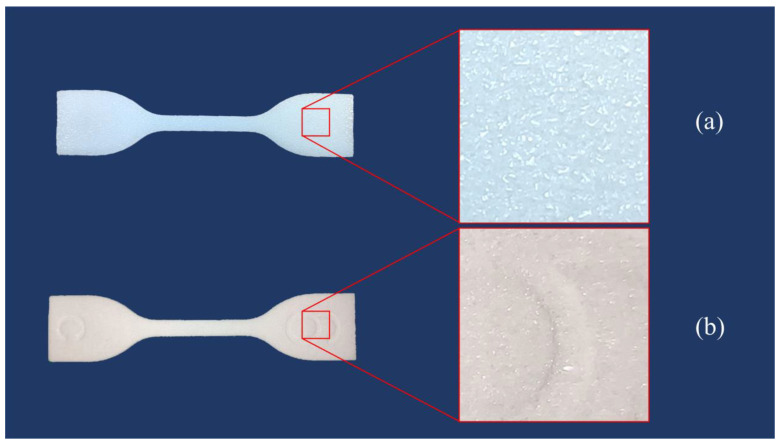
Comparison of specimen surfaces: (**a**) specimen with post-processing and (**b**) specimen without post-processing.

During post-processing, the chemical solvent would react with the surface of the printed part, which may change the composition of the part. Therefore, it is necessary to study the effect of post-processing on the mechanical properties of 3D printed TPU parts. In this study, the chemical treatment was performed by immersing the printed part into an amide solvent for 3 min. Figure 5 shows the surfaces of the specimens with and without post-processing. As can be seen, the specimen with post-processing has a white and smooth surface, whereas the specimen without post-processing has a yellowish-white and grainy surface.

#### 2.2.3. Powder Mix Ratio

After SLS processing, the un-sintered powder was often recycled and reused. As TPU particles may stick together during the SLS process, the large particles were filtered out from the un-sintered powder using a sieve with a mesh size of 0.178 mm. However, the temperature in the SLS machine may affect the thermoplastic properties of the un-sintered powder. In this study, three mix ratios commonly utilized in industry are investigated: 30% new powder and 70% reused powder, 50% new powder and 50% reused powder, and 100% new powder. A total of 8 sets of specimens were printed with ten specimens in each set. Details of the test specimens are summarized in Table 3.

### 2.3. Tensile Test Setup

**Figure 6:**
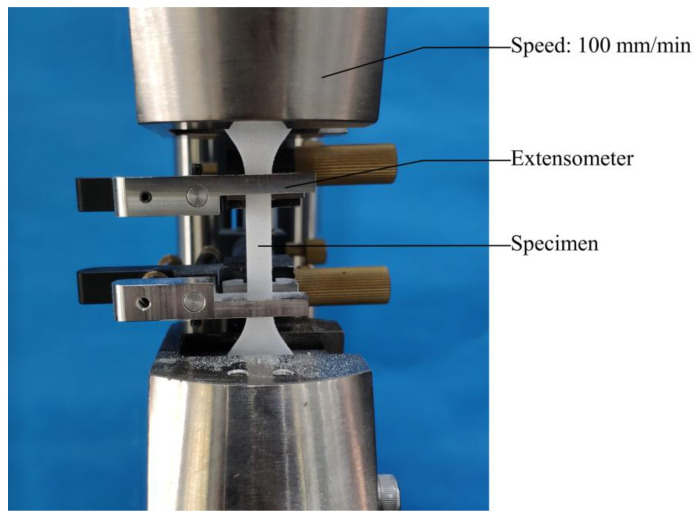
Tensile test setup.

A 10 kN universal testing machine with a 250 N load cell was used for the tensile tests. The elongation of the length of the specimen was measured by an extensometer. During the test, a small prestress (about 2 N) was applied to the specimen to avoid bending. The tensile load was applied under displacement control at a constant rate of 100 mm/min (Figure 6).

### 2.4. Scanning Electron Microscope (SEM)

According to the literature [41,42], the ideal shape of the TPU powder is plump and spherical. It is believed that a better powder shape could improve the quality of the printed parts. In this study, a scanning electron microscope (SEM, Tescan-MIRA 3, Kohoutovice, Czech Republic) was used for shape analysis to check the quality of the new powder and the reused powder.

### 2.5. Particle-Size Distribution (PSD)

Particle-size distribution is an index indicating the proportion of particle sizes in a sample particle group. As particle size can influence the print process during SLS, PSD is important for the understanding of TPU powder [43]. One of the most common methods of measuring the PSD is sieve analysis (or gradation test), which contains a series of sieves with decreasing mesh sizes. The particles pass through the sieves and the weight of material that is maintained at each level of sieving is measured. In this study, a sieve analysis (Mastersizer-2000, Malvern, UK) was carried out to obtain quantitative information about the difference in particle size distribution between the new powder and the reused powder.

### 2.6. Melt Flow Rate (MFR)

MFR is a parameter which indicates the ease of the flow of the melted thermoplastics. It determines the viscosity and quality of the thermoplastics. To obtain MFR, the melted TPU material would flow through a specific capillary at a prescribed temperature and pressure, and the weight of the material flowing through the capillary within 10 min is measured (melt flow indexer: XNR-400A, Qingdao, China). According to ASTM standard 1238 (Standard test method for melt flow rates of thermoplastics) [44], the test conditions for TPU are 230 °C/2.16 kg.

### 2.7. Differential Scanning Calorimetry (DSC)

**Figure 7:**
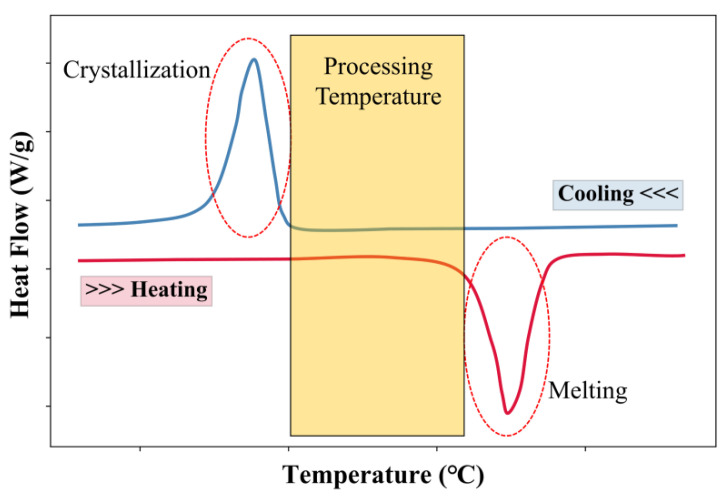
Typical differential scanning calorimetry (DSC) traces of thermoplastic polyurethane (TPU) material.

As illustrated in Figure 1, a laser beam is used to selectively sinter the TPU powder deposited in a thin layer during SLS processing. The heater in the SLS machine provides an appropriate processing temperature (ambient temperature) to suppress the crystallization of the powder melted by the laser as long as possible so that the sintered layers could better adhere to the next processing layer. Differential scanning calorimetry (DSC, Mettler Toledo-Q2000, Columbus, OH, USA) could be used to measure the thermal properties of the TPU powder. Figure 7 shows the typical DSC traces of TPU material. The melting peak during heating and the crystallization peak during cooling are marked (red circles) in the figure, and the ideal processing temperature (yellow area) is between melting and crystallization. In this study, the thermal properties of both the new powder and the reused powder were measured by DSC. The tests were carried out under a nitrogen atmosphere to simulate the environment of SLS processing. The powder was heated from 30 °C at a rate of 50 °C/min, and then cooled from 240 to −70 °C at a rate of 10 °C/min.

## 3. Results and Discussion

### 3.1. Effect of Build Orientation

**Figure 8:**
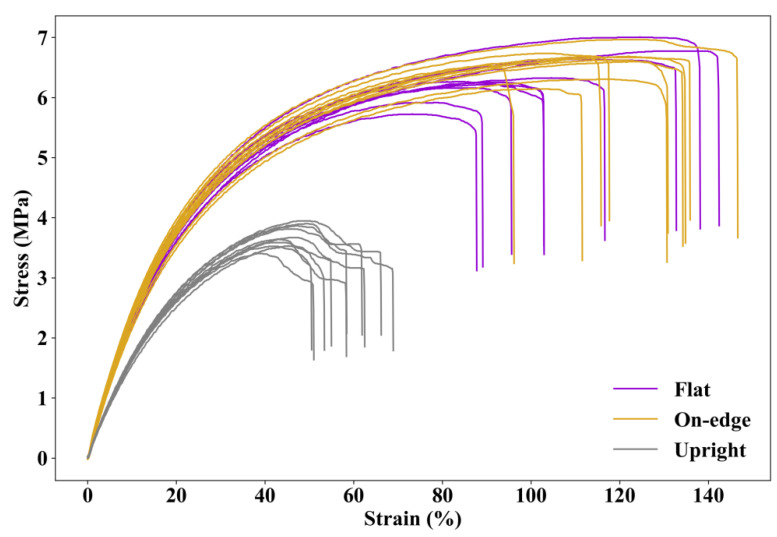
Stress–strain curves of specimens printed in three different directions.

The stress–strain relationships obtained from the tensile tests for sets 1, 2 and 3 are compared in Figure 8. The averaged maximum tensile strength (TS) and the strain at break ($\varepsilon_{b}$) are tabulated in Table 4.

**Figure 9:**
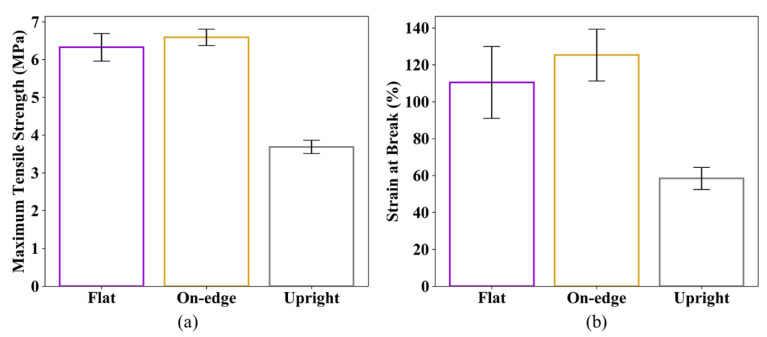
Average and standard deviation of the results: (**a**) Maximum tensile strength and (**b**) strain at break.

As can be seen in Figure 8, all stress–strain curves demonstrate a non-linear behavior of SLS printed TPU. The average maximum tensile strengths of specimens printed in flat and on-edge orientations are similar (6.32 MPa and 6.65 MPa, respectively). The specimens printed in on-edge orientation have a larger strain capacity with an average strain at break of 125.27%, whereas it is 110.46% for specimens printed in flat orientation. Compared to flat and on-edge orientations, the specimens printed in upright orientation show a significant drop in both tensile strength and strain capacity. The average maximum tensile strength of the specimens printed in upright orientation is only 3.69 MPa, and the strain at break is 58.43%. Figure 9 presents the average and standard deviation of the maximum tensile strength and strain at break obtained from the experimental tests. It is worth noting that the samples printed in flat orientation show larger variations in the test results than the others.

**Figure 10:**
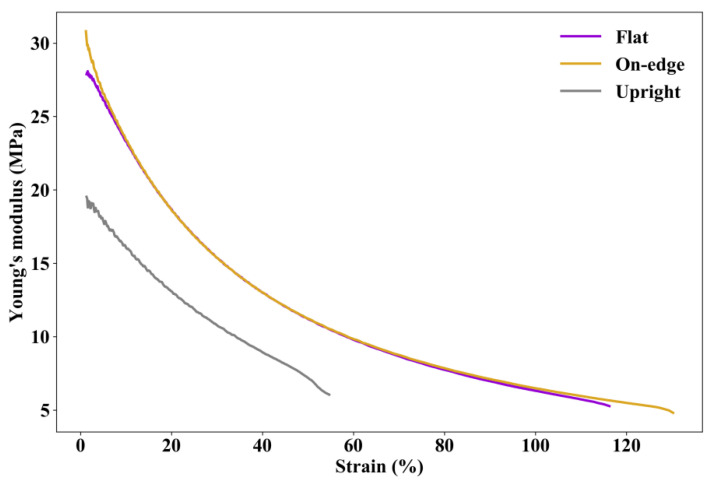
Moduli at different strains of specimens printed in different orientations.

Since TPU is highly non-linear even at a low strain level, the moduli of printed TPU specimens at different strains are calculated and shown in Figure 10. It can be seen that the moduli of specimens printed in flat and on-edge orientations are very close to slightly larger values for the specimen printed in on-edge orientation when the strain is at extremely small and extremely large levels. The modulus of specimen printed in upright orientation is significantly lower than those in flat orientation and on-edge orientation.

**Figure 11:**
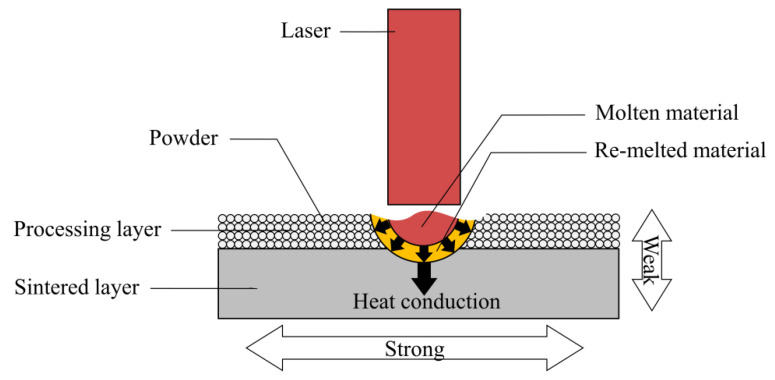
Illustration of selective laser sintering (SLS) processing.

The tensile test results are consistent with the studies where the specimens were printed using the fused deposition modeling (FDM) technique [34]. The lower tensile strength, stiffness and deformability obtained for the specimens printed in upright orientation are attributed to the weak bond between the printed layers. In this study, the laser melts the powder in the processing layer, as illustrated in Figure 11. As the heat is conducted from the processing layer to the sintered layer, a small area of the sintered part is re-melted and bonded with the molten material. Thus, the interlayer bonding strength is much higher than the bonding strength between the layers. In the tensile test, the specimens printed in upright orientation are pulled in a direction perpendicular to the layers, leading to premature bond failure.

### 3.2. Effect of Post-Processing

**Figure 12:**
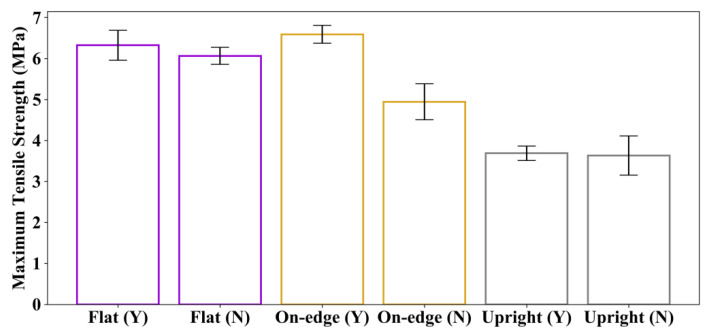
Comparison of the average maximum tensile strength. “Y” indicates specimens with post-processing, and “N” means specimens without post-processing.

**Figure 13:**
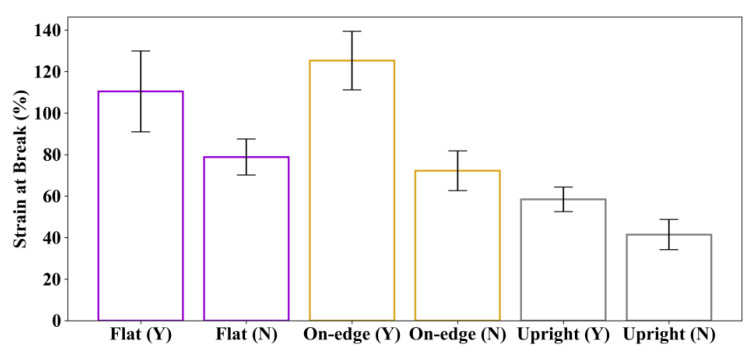
Comparison of the average strain at break. “Y” indicates specimens with post-processing, and “N” means specimens without post-processing.

The average tensile test results of the specimens without post-processing (sets 4, 5 and 6) and those with post-processing (sets 1, 2 and 3) are summarized in Table 5, and compared in Figure 12 and Figure 13.

Figure 12 shows the comparison of the average maximum tensile strength of the specimens with and without post-processing. As can be seen, post-processing has almost no effect on the tensile strength of specimens printed in flat and upright orientations. The specimens printed in flat orientation have the least number of layers, and the load is parallel to the layers, so the tensile strength mainly depends on the strength of each layer. The specimens printed in upright orientation have the largest number of layers, and the load is perpendicular to the layers. Hence, the tensile strength mainly depends on the bond strength between layers. As post-processing does not affect the strength of each layer and the bond strength between layers, its effect on the tensile strength of specimens printed in flat and upright orientations is very limited. As for the specimens printed in on-edge orientation, the tensile strength is increased by 34.6% compared to those without post-processing. This is because the specimens printed in on-edge orientation have more layers than those printed in flat orientation. Although the applied load is parallel to the layers during testing, delamination may occur due to the small angle between the load and the layers, which could cause premature failure of the specimens. In this case, the tension of the coating formed by the post-processing can suppress delamination, thereby increasing the tensile strength of the specimen.

The comparison of the average strain at break of the specimens with and without post-processing is illustrated in Figure 13. It can be seen that the strains at break are considerably increased for specimens with post-processing. The strains at break are increased by 40.2%, 73.5% and 41.0%, respectively, for post-processed specimens printed in flat, on-edge and upright orientations.

**Figure 14:**
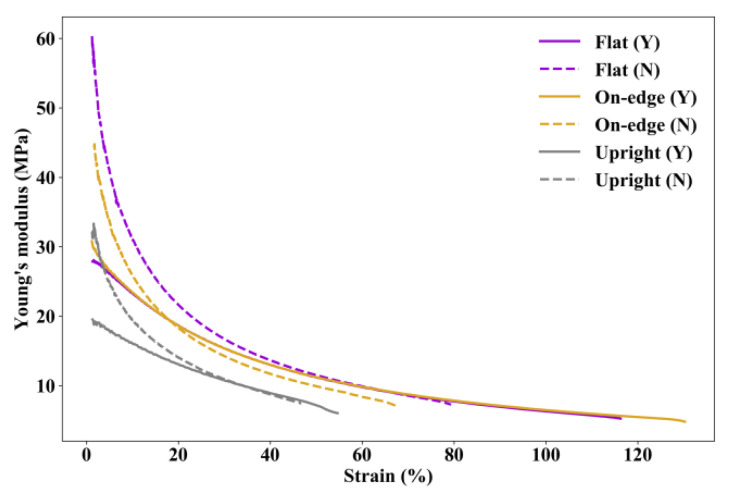
Comparison of the moduli at different strains of specimens. “Y” indicates specimens with post-processing, and “N” means specimens without post-processing.

Figure 14 shows the comparison of the moduli of specimens with and without post-processing. Since post-processing increases the deformability but has little influence on the tensile strength of TPU, the moduli of specimens are reduced. Especially, when the strain is at a relatively low level, an obvious discrepancy can be observed between the specimens with and without post-processing. However, it shall also be noted that post-processing could generate a relatively constant Young’s modulus at different strain levels.

### 3.3. Effect of Powder Mix Ratio

**Figure 15:**
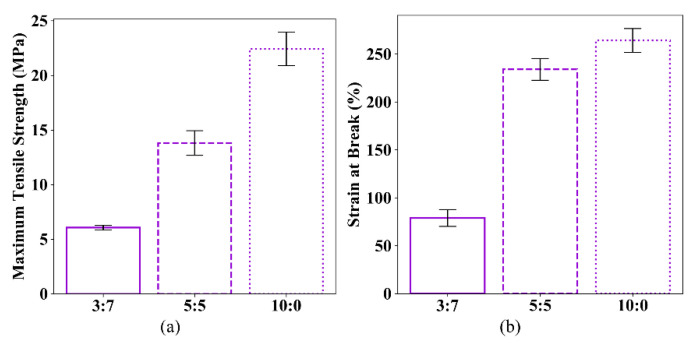
Comparison of (**a**) maximum tensile strength and (**b**) strain at break.

The average tensile test results of the specimens with different powder mix ratios (sets 4, 7 and 8) are listed in Table 6. All these specimens are printed in flat orientation without post-processing. Figure 15 shows the comparison of the tensile test results of specimens with three different mix ratios.

It can be seen from Figure 15 that the mechanical behavior of specimens is dramatically influenced by the mix ratio of the new powder and reused powder. Compared with the specimens printed with 30% new powder and 70% reused powder, the maximum tensile strength and the strain at break of the specimens printed with 50% new powder and 50% reused powder are increased by 127.9% and 196.8%, respectively. The maximum tensile strength and the strain at break of the specimens printed with 100% new powder are increased by 270.0% and 235.1%, respectively. In conclusion, the higher the proportion of the new powder, the better the mechanical performance of the printed parts, however, the cost is also higher.

**Figure 16:**
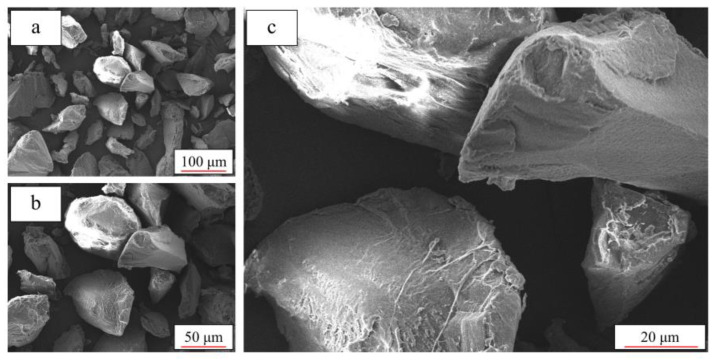
Microstructure of new TPU powder under different magnifications: (**a**) 500×; (**b**) 1000× and (**c**) 2000×.

**Figure 17:**
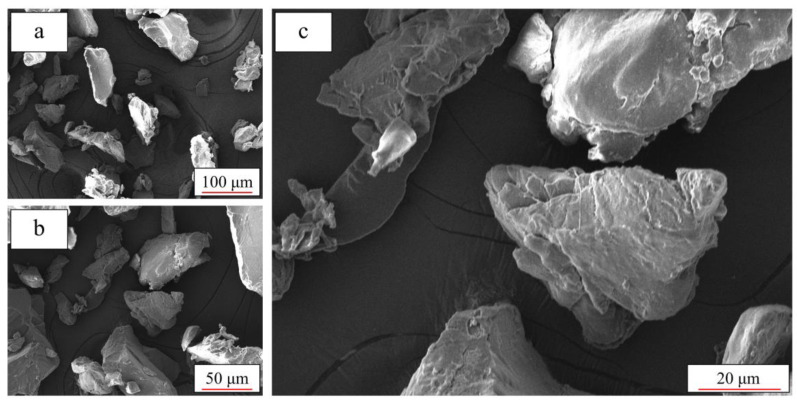
Microstructure of reused TPU powder under different magnifications: (**a**) 500×; (**b**) 1000× and (**c**) 2000×.

In fact, the mechanical behavior of additively manufactured TPU parts is closely related to the particle quality and thermal behavior of the powder [29]. Figure 16 and Figure 17 show the selected SEM images of the new powder and the reused powder. It can be seen in Figure 16 that the new TPU powder contains plump particles with smooth surfaces and clear edges, whereas the particles of the reused powder are shrunken (Figure 17), and they have rough surfaces and vague edges. Obviously, the new powder has better particle quality compared to the reused powder.

**Figure 18:**
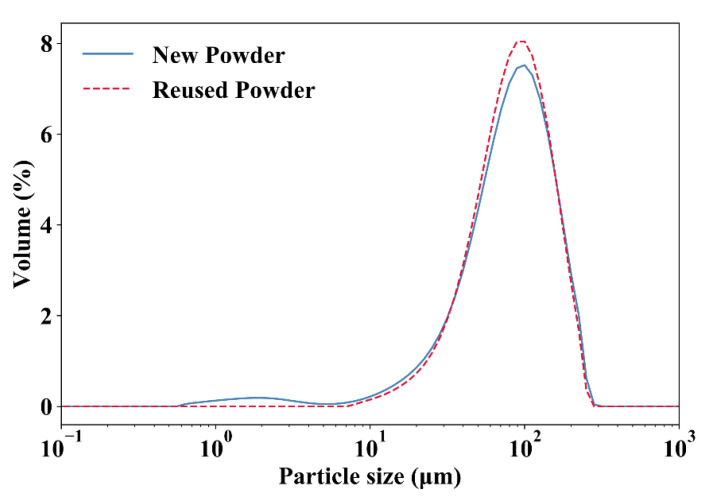
Particle size distribution results for the new powder and the reused powder.

Figure 18 shows the particle size distributions for the new powder and the reused powder. As can be seen, no apparent difference in particle size is observed between the new powder and the reused powder. However, it is worth noting that there is a small peak at about 1 µm on the curve of the new powder, and the volume-weighted mean diameter of the reused powder (88.08 µm) is slightly larger than the new powder (86.87 µm). This may be due to a small amount of solid-state diffusion that took place (smaller particles fuse together to become larger particles) under the bed temperature.

The flow rate values for the new powder and the reused powder are 74 g/10 min and 71 g/10 min, respectively. The results show that the new powder’s flow rate value is marginally higher than the reused powder, which means that it is easier for the new powder to be formed into the printed part.

**Figure 19:**
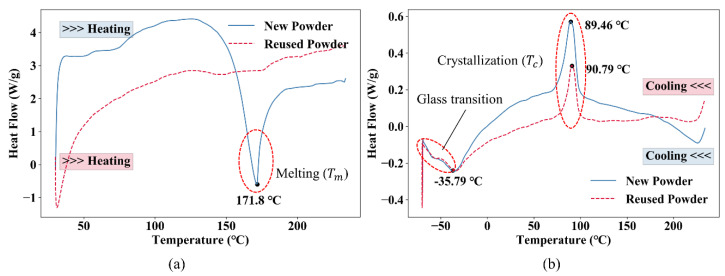
Differential scanning calorimetry (DSC) traces of the new powder and the reused powder: (**a**) heating traces and (**b**) cooling traces.

Figure 19 presents the DSC traces of the new powder and the reused powder. The heating trace of the new powder shows a melting peak at around 171.8 °C, whereas no clear melting peak is observed in the heating trace of the reused powder. The cooling traces of the new powder and the reused powder show similar trends, with crystallization temperatures at 89.5 °C and 90.8 °C, respectively. The glass transitions of the new powder and the reused powder are almost the same. It can be seen that the effect of mix ratio mainly depends on whether the powder could be melted easily. Since the new powder has a distinct melting peak on its heating curve, it is easy to melt under laser irradiation. Therefore, the proportion of new powder should be increased if the products require better mechanical properties.

The similar particle size distributions and flow rate values obtained for the new powder and the reused powder demonstrate that the PSD and MFR have limited influence on the mechanical properties of TPU specimens; whereas finer particle quality and proper thermal behavior could give rise to better mechanical properties of the printed TPU structures.

## 4. Conclusions

In this paper, the material properties of thermoplastic polyurethane (TPU) specimens additively manufactured using an industrial selective laser sintering (SLS) machine are studied. In particular, the effects of build orientation, powder mix ratio and post-processing on the mechanical properties of 3D printed TPU specimens are systematically investigated. The main findings from this study are as follows.

- The most significant way to improve the mechanical properties of printed parts is to increase the mix ratio of the new powder. The new powder has better particle quality and thermal properties, which are more effective for SLS processing.
- TPU specimens printed in flat and on-edge orientations exhibit similar superior mechanical properties. For the specimens printed in upright orientation, the tensile strength is about 40% lower and the deformability is about 60% lower compared to the other two orientations.
- Post-processing can substantially enhance the deformability of the specimens. For flat, on-edge and upright orientations, the deformability can be increased by 40.1%, 73.5% and 41.0%, respectively.

Results from this study provide much-needed experimental data for analyzing and optimizing products made of additively manufactured TPU.

## Figures and Tables

**Table 1:** Dimensions of the dumb-bell specimens [40].

Dimension	Value (mm)
entry 1	data
A. Overall length (minimum)	115
B. Width of ends	25 ± 1
C. Length of the narrow portion	33 ± 2
D. Width of the narrow portion	6.2 ± 0.2
E. Transition radius outside	14 ± 1
F. Transition radius inside	25 ± 2
Test length	25 ± 0.5
Thickness	2 ± 0.2

**Table 2:** Processing parameters used in this study.

Parameters	Value
Build orientation	Flat, on-edge, upright
Post-processing method	Chemical treatment, none
Powder mix ratio	
(new powder: reused powder)	3:7; 5:5; 10:0

**Table 3:** Eight sets of specimens printed with different processing parameters.

Set Number	Build Orientation	Post-Processing Method	Powder Mix Ratio (New Powder: Reused Powder)
1	Flat	Chemical treatment	3:7
2	On-edge	Chemical treatment	3:7
3	Upright	Chemical treatment	3:7
4	Flat	None	3:7
5	On-edge	None	3:7
6	Upright	None	3:7
7	Flat	None	5:5
8	Flat	None	10:0

**Table 4:** Average tensile test results of the specimens.

Set Number	Build Orientation	TS (MPa)	$\varepsilon_{b}\;\left(\%\right)$
1	Flat	6.32	110.46
2	On-edge	6.65	125.27
3	Upright	3.69	58.43

**Table 5:** Average tensile test results of specimens with and without post-processing.

Set	Build Orientation	Post-Processing Method	TS (MPa)	$\varepsilon_{b}\;\left(\%\right)$
1	Flat	Chemical treatment	6.32	110.46
2	On-edge	Chemical treatment	6.65	125.27
3	Upright	Chemical treatment	3.69	58.43
4	Flat	None	6.06	78.81
5	On-edge	None	4.94	72.20
6	Upright	None	3.63	41.45

**Table 6:** Average tensile test results of the specimens.

Set Number	Mix Ratio (New Powder: Reused Powder)	TS (MPa)	$\varepsilon_{b}\;\left(\%\right)$
4	3: 7	6.06	78.81
7	5: 5	13.81	233.91
8	10: 0	22.42	264.07

## References

[1] Bikas H., Stavropoulos P., Chryssolouris G. (2016). Additive manufacturing methods and modeling approaches: A critical review. Int. J. Adv. Manuf. Technol..

[2] Hague R.J.M. (2006). Unlocking the design potential of rapid manufacturing. Rapid Manufacturing: An Industrial Revolution for the Digital Age.

[3] Xie Y.M., Steven G.P. (1993). A simple evolutionary procedure for structural optimization. Comput. Struct..

[4] Xiong Y., Yao S., Zhao Z.L., Xie Y.M. (2020). A new approach to eliminating enclosed voids in topology optimization for additive manufacturing. Addit. Manuf..

[5] Zegard T., Paulino G.H. (2016). Bridging topology optimization and additive manufacturing. Struct. Multidiscip. Optim..

[6] Wang X., Xu S., Zhou S., Xu W., Leary M., Choong P., Qian M., Brandt M., Xie Y.M. (2016). Topological design and additive manufacturing of porous metals for bone scaffolds and orthopaedic implants: A review. Biomaterials.

[7] He Y., Cai K., Zhao Z.L., Xie Y.M. (2020). Stochastic approaches to generating diverse and competitive structural designs in topology optimization. Finite Elem. Anal. Des..

[8] Bi M., Tran P., Xie Y.M. (2020). Topology optimization of 3D continuum structures under geometric self-supporting constraint. Addit. Manuf..

[9] Calignano F., Manfredi D., Ambrosio E.P., Biamino S., Lombardi M., Atzeni E., Salmi A., Minetola P., Iuliano L., Fino P. (2017). Overview on additive manufacturing technologies. Proc. IEEE.

[10] Kruth J.P., Mercelis P., Van Vaerenbergh J., Froyen L., Rombouts M. (2005). Binding mechanisms in selective laser sintering and selective laser melting. Rapid Prototyp. J..

[11] Agarwala M., Bourell D., Beaman J., Marcus H., Barlow J. (1995). Direct selective laser sintering of metals. Rapid Prototyp. J..

[12] Lamikiz A., Sánchez J.A., López de Lacalle L.N., Arana J.L. (2007). Laser polishing of parts built up by selective laser sintering. Int. J. Mach. Tools Manuf..

[13] Barroqueiro B., Andrade-Campos A., Valente R.A.F., Neto V. (2019). Metal additive manufacturing cycle in aerospace industry: A comprehensive review. J. Manuf. Mater. Process..

[14] Berry E., Brown J.M., Connell M., Craven C.M., Efford N.D., Radjenovic A., Smith M.A. (1997). Preliminary experience with medical applications of rapid prototyping by selective laser sintering. Med. Eng. Phys..

[15] Liu Z., Zhang P., Yan M., Xie Y.M., Huang G. (2019). Additive manufacturing of specific ankle-foot orthoses for persons after stroke: A preliminary study based on gait analysis data. Math. Biosci. Eng..

[16] Mărieş G.R.E., Bandur G., Rusu G. (2008). Influence of processing temperature on some mechanical-physical properties of thermoplastic polyurethane desmopan KA 8377 used for injection moulding of performance sport products. Chem. Bull. Politeh. Univ. (Timişoara).

[17] Aurilia M., Piscitelli F., Sorrentino L., Lavorgna M., Iannace S. (2011). Detailed analysis of dynamic mechanical properties of TPU nanocomposite: The role of the interfaces. Eur. Polym. J..

[18] Ma H., Yang Y. (2008). Rheology, morphology and mechanical properties of compatibilized poly(vinylidene fluoride) (PVDF)/thermoplastic polyurethane (TPU) blends. Polym. Test..

[19] Li W., Liu J., Hao C., Jiang K., Xu D., Wang D. (2008). Interaction of thermoplastic polyurethane with polyamide 1212 and its influence on the thermal and mechanical properties of TPU/PA1212 blends. Polym. Eng. Sci..

[20] Mi H.-Y., Salick M.R., Jing X., Jacques B.R., Crone W.C., Peng X.-F., Turng L.-S. (2013). Characterization of thermoplastic polyurethane/polylactic acid (TPU/PLA) tissue engineering scaffolds fabricated by microcellular injection molding. Mater. Sci. Eng. C.

[21] Poomali, Siddaramaiah, Suresha B., Lee J.-H. (2008). Mechanical and three-body abrasive wear behaviour of PMMA/TPU blends. Mater. Sci. Eng. A.

[22] Feng F., Ye L. (2011). Morphologies and mechanical properties of polylactide/thermoplastic polyurethane elastomer blends. J. Appl. Polym. Sci..

[23] Lu Q.W., Macosko C.W., Horrion J. (2003). Compatibilized blends of thermoplastic polyurethane (TPU) and polypropylene. Macromol. Symp..

[24] Lee H., Eom R.I., Lee Y. (2019). Evaluation of the mechanical properties of porous thermoplastic polyurethane obtained by 3D printing for protective gear. Adv. Mater. Sci. Eng..

[25] Bates S.R.G., Farrow I.R., Trask R.S. (2016). 3D printed polyurethane honeycombs for repeated tailored energy absorption. Mater. Des..

[26] Bates S.R.G., Farrow I.R., Trask R.S. (2016). 3D printed elastic honeycombs with graded density for tailorable energy absorption. Active and Passive Smart Structures and Integrated Systems.

[27] Scarpello M.L., Kazani I., Hertleer C., Rogier H., Vande Ginste D. (2012). Stability and efficiency of screen-printed wearable and washable antennas. IEEE Antennas Wirel. Propag. Lett..

[28] Li Y., Zhou B., Zheng G., Liu X., Li T., Yan C., Cheng C., Dai K., Liu C., Shen C. (2018). Continuously prepared highly conductive and stretchable SWNT/MWNT synergistically composited electrospun thermoplastic polyurethane yarns for wearable sensing. J. Mater. Chem. C.

[29] Dadbakhsh S., Verbelen L., Vandeputte T., Strobbe D., Van Puyvelde P., Kruth J.P. (2016). Effect of powder size and shape on the SLS processability and mechanical properties of a TPU elastomer. Phys. Procedia.

[30] Gan X., Wang J., Fei G., Xia H. (2019). Selective laser sintering of polyurethane elastomers. Polym. Mater. Sci. Eng..

[31] Verbelen L., Dadbakhsh S., Van den Eynde M., Strobbe D., Kruth J.P., Goderis B., Van Puyvelde P. (2017). Analysis of the material properties involved in laser sintering of thermoplastic polyurethane. Addit. Manuf..

[32] Yuan S., Shen F., Bai J., Chua C.K., Wei J., Zhou K. (2017). 3D soft auxetic lattice structures fabricated by selective laser sintering: TPU powder evaluation and process optimization. Mater. Des..

[33] Barba D., Alabort C., Tang Y.T., Viscasillas M.J., Reed R.C., Alabort E. (2020). On the size and orientation effect in additive manufactured Ti-6Al-4V. Mater. Des..

[34] Chacón J.M., Caminero M.A., García-Plaza E., Núñez P.J. (2017). Additive manufacturing of PLA structures using fused deposition modelling: Effect of process parameters on mechanical properties and their optimal selection. Mater. Des..

[35] Ramos J.A., Bourell D.L. (2002). Modeling of surface roughness enhancement of indirect-SLS metal parts by laser surface polishing. Proc. TMS Fall Meet..

[36] Boualleg A. (2019). Investigations on Post-Processing of 3D Printed Thermoplastic Polyurethane (TPU) Surface.

[37] Allen K.W. (1997). Polymer Surface Modification: Relevance to Adhesion.

[38] Molitor P., Barron V., Young T. (2001). Surface treatment of titanium for adhesive bonding to polymer composites: A review. Int. J. Adhes. Adhes..

[39] Kumar S. (2003). Selective laser sintering: A Qualitative and objective approach. JOM.

[40] International Organization for Standardization ISO 37: Rubber, Vulcanized or Thermoplastic—Determination of Tensile Stress-Strain Properties. https://www.iso.org/standard/68116.html.

[41] Cheng J., Lao S., Nguyen K., Ho W., Cummings A., Koo J. SLS processing studies of nylon 11 nanocomposites. Proceedings of the 16th Solid Freeform Fabrication Symposium, SFF.

[42] Schmid M., Wegener K. (2016). Additive Manufacturing: Polymers applicable for laser sintering (LS). Proc. Eng..

[43] Plummer K., Vasquez M., Majewski C., Hopkinson N. (2012). Study into the recyclability of a thermoplastic polyurethane powder for use in laser sintering. Proc. Inst. Mech. Eng. Part B J. Eng. Manuf..

[44] ASTM International ASTM D1238-20: Standard Test Method for Melt Flow Rates of Thermoplastics by Extrusion Plastometer. http://www.astm.org/cgi-bin/resolver.cgi?D1238.

